# Supplementary figures and images for: Yth m6A RNA-Binding Protein 1 Regulates Osteogenesis of MC3T3-E1 Cells under Hypoxia via Translational Control of Thrombospondin-1
Source: Int J Mol Sci. 2023 Jan 16;24(2):1741. doi: 10.3390/ijms24021741 (PMC9863954; doi:10.3390/ijms24021741)

**Normoxia**

**NC-oe**

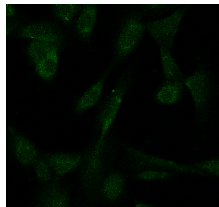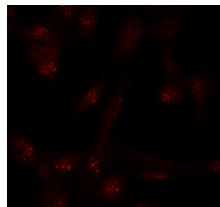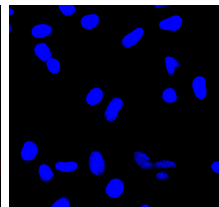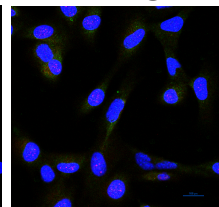

**Ythdf1-oe**

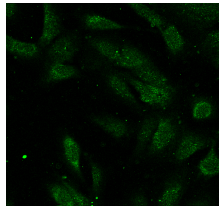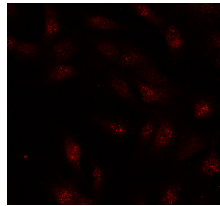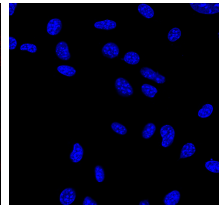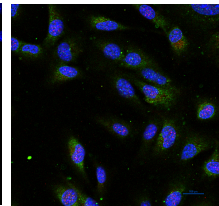

**Hypoxia**

**NC-oe**

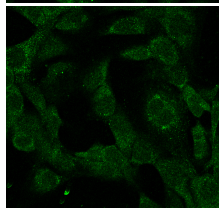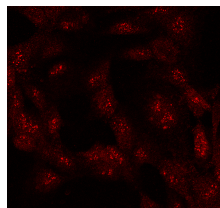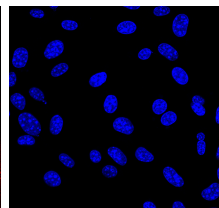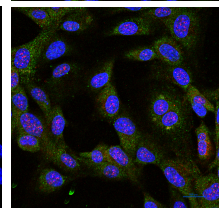

**Ythdf1-oe**

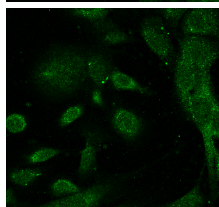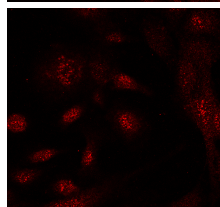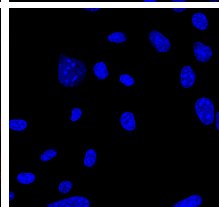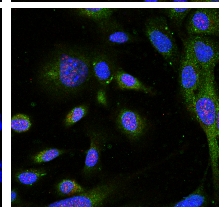

Supplement: Supplementary file 1 [file ijms-24-01741-s001.zip › ijms-2121691-supplementary.pdf]
